# Pre-Analytical Determination of the Effect of Extended Warm or Cold Ischaemia on RNA Stability in the Human Ileum Mucosa

**DOI:** 10.1371/journal.pone.0138214

**Published:** 2015-09-15

**Authors:** Serene M. L. Lee, Celine Schelcher, Reinhard Thasler, Tobias S. Schiergens, Wolfgang E. Thasler

**Affiliations:** 1 Department of General, Visceral, Transplantation, Vascular and Thoracic Surgery, Hospital of the University of Munich, Munich, Germany; 2 Biobank under the administration of the Human Tissue and Cell Research (HTCR) Foundation, Department of General, Visceral, Transplantation, Vascular and Thoracic Surgery, Hospital of the University of Munich, Munich, Germany; University of Palermo, ITALY

## Abstract

The use of banked human tissue, obtained with informed consent after elective surgical procedures, represents a powerful model for understanding underlying mechanisms of diseases or therapeutic interventions and for establishing prognostic markers. However, donated tissues typically have varying times of warm ischaemia *in situ* due to blood arrest or cold ischaemia due to procurement and transportation. Hence, before using these tissues, it is important to carry out pre-analytical studies to ensure that they are representative of the *in vivo* state. In particular, tissues of the gastrointestinal tract have been thought to have low RNA stability. Therefore, this study aimed to determine if extended warm or cold ischaemia times and snap-freezing or banking in RNA stabilization solution affects RNA integrity or gene expression in human ileum mucosa. In short, ileum mucosa was collected for up to 1.5 h and 6 h of simulated warm or cold ischaemia respectively. Subsequently, RNA integrity and gene expressions were determined. It was found that RNA integrity remained high over the course of warm and cold ischaemia examined and there were in general no significant differences between snap-freezing and banking in RNA stabilization solution. Following the same trend, there were in general no significant changes in gene expressions measured (*MYC*, *HIF1α*, *CDX*, *HMOX1* and *IL1β*). In conclusion, RNA in the ileum mucosa is maintained at a high integrity and has stable gene expression over the examined time course of warm or cold ischaemia when banked in RNA stabilization solution or snap-frozen in liquid nitrogen. As the average warm and cold ischaemia times imposed by surgery and the process of tissue banking are shorter than the time period examined in this study, human ileum mucosa samples collected after surgeries could be used for gene expression studies.

## Introduction

Comparative quantification of gene expression, through real-time quantitative polymerase chain reaction (RT-qPCR) or gene expression microarrays, is essential for understanding the molecular bases of diseases. For such studies, human tissue banks such as the one at the Surgical Clinic in the Hospital of the University of Munich, provide powerful models by banking leftover tissue in excess of what is required for diagnostic tests by the pathologists. For these tissues to provide relevant experimental results, the banked tissue must be of a high quality representative of the *in vivo* state. Unlike the use of animal models, human tissue is typically obtained from resected tissues from elective surgeries after obtaining informed consent from the donor. As such, there will be varying warm and cold ischaemia times associated with each tissue dependent on the blood arrest time during the operation or the transport time from the operation suite to the pathologist and finally to the tissue bank [1].

Studies have been undertaken by various investigators to examine the effect of temperature, time and banking method (snap-freezing/ banking in RNA stabilization solution) on RNA integrity and gene expression in a variety of tissues (S1 and S2 Tables). For the gut in particular, investigators have examined RNA quality and gene expression in the duodenum [2] and colon [3, 4]. However, to our knowledge, the effects of the above conditions on ileum mucosa have not been examined. This is of interest as previous studies have shown that RNA was least stable in tissues of the gastrointestinal tract [2, 5] possibly due to the presence of ribonucleases (RNases), which play a role in epithelial host defence [6–9].

For ileum collected in this hospital, the average warm ischaemia time (37°C) was 12 ± 13 min and the average cold ischaemia time (on ice) was 22 ± 15 min with values expressed as means ± standard deviation (*N* = 52). Therefore, this study aimed to examine the effects of extended warm or cold ischaemia time and banking method (snap-freezing in liquid nitrogen or banking in RNA stabilization solution) on RNA integrity and gene expression.

## Materials and Methods

### Human ileum collection

The tissues and data used in this study were provided by the Biobank (http://www.klinikum.uni-muenchen.de/Chirurgische-Klinik-und-Poliklinik-Grosshadern/de/0800-gewebebank/index.html) located in the Hospital of the University of Munich, which operates in accordance with the European Union-compliant ethical and legal framework of the Human Tissue and Cell Research (HTCR) Foundation (http://www.htcr.org) [10]. The process of tissue collection included obtaining written informed consent from all 6 donors. This framework has also been approved by the ethics commission of the Faculty of Medicine in the University of Munich and the Bavarian State Medical Association.

From each donor with colon carcinoma, a piece of terminal ileum was collected in the operation room during right hemicolectomy by the staff from the Biobank. During the surgical procedure, the terminal ileum part of the preparation was dissected out after the detachment of the branches of the middle colic artery and the right colic artery and before the complete resection of the whole preparation. The terminal ileum was dissected as follows. While the vascular structure remains intact, a 5 cm length of terminal ileum was isolated between two staplers. The vascular pedicle still supplying this part of the ileum was then clamped using an Overholt and the isolated ileum was then immediately separated and put on a back table for the preparation of ileum mucosa samples by the staff from the Biobank. The surgeon then completes the surgical intervention and removal of the remaining hemicolectomy preparation. During this procedure, no additional ileum tissue had to be resected as only the piece of terminal ileum, which is part of the hemicolectomy preparation, was separated in situ by slightly adapting surgical procedures with no effect on the therapeutic intervention.

**Fig 1:**
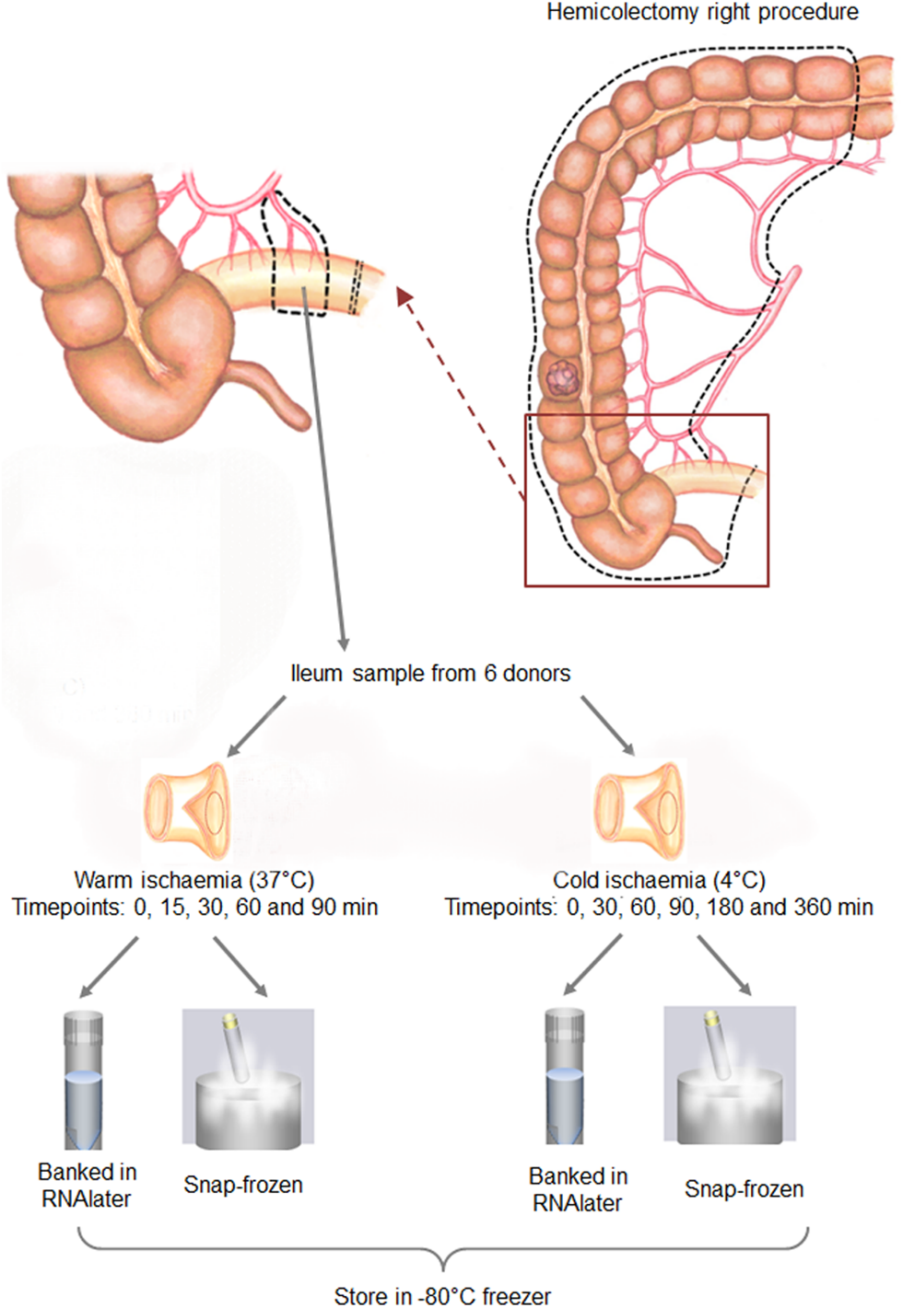
Experimental plan for the collection of human ileum mucosa specimens from hemicolectomy right surgeries to examine the effects of warm or cold ischaemia and different processing methods used before banking of the tissues at -80°C.

When the staff from the Biobank obtained the terminal ileum in the operation room, warm and cold ischaemia times remained less than 1 minute. As such, samples collected immediately were labeled as “time 0”. The whole ileum mucosa collection procedure is shown in Fig 1. In short, the piece of tissue collected from each donor was halved with one piece placed at 37°C to simulate warm ischemia (for 15, 30, 60 and 90 min) and the other at 4°C to simulate cold ischemia (for 30 min, 60 min, 3h and 6 h) respectively. At each time point for warm or cold ischaemia, two 30 mg pieces of ileum mucosa were sampled, one was snap-frozen and the other was banked using RNAlater RNA Stabilization Reagent (Qiagen, Hilden, Germany) according to manufacturer’s instructions.

### H & E staining of ileum sections

FFPE ileum samples were sectioned at 6 μm thickness. These sections were stained according to the instructions for use that came with the Mayer’s haematoxylin solution (Merck, Hessen, Germany) with some modifications. The modifications were that the slides were rinsed in 1% HCl in 70% ethanol for 2 seconds after haematoxylin staining and a dip in distilled water was done after each rinse in running tap water before the next step.

### Extraction and quantification of RNA from the ileum mucosa

The extraction of RNA from the ileum mucosa was performed using the RNeasy Mini Kit (Qiagen, Hilden, Germany) according to the manufacturer’s protocol. In brief, 30 mg of ileum mucosa was homogenised in the provided lysis buffer using the TissueLyser LT bead mill (Qiagen, Hilden, Germany) according to the manufacturer’s protocol. The homogenate was then transferred into a RNeasy spin column, which binds RNA using a silica-membrane. In order to remove any potential DNA contamination, an on-column DNA digestion step was done using the RNase-free DNase Set (Qiagen, Hilden, Germany). The bound RNA was finally eluted from the spin column in 30 μl of RNase free water.

Assessment of nucleic acid purity was done using the NanoDrop 2000 spectrophotometer (Thermo Scientific, Wilmington, USA). To quantify RNA, the Quant-iT Ribogreen RNA Assay Kit (Life Technologies, Darmstadt, Germany) was used according to the manufacturer’s protocol. RNA was quantitated with a fluorescent nucleic acid stain that can be detected using the Filtermax F3 microplate reader (Molecular Devices, Biberach an der Riss, Germany) operating with excitation / emission wavelengths of 480 nm / 520 nm respectively.

### Determination of the RNA integrity

To determine the RNA integrity, 200 ng of RNA from each sample was loaded on the RNA 6000 Nano Chip (Agilent Technologies, Waldbronn, Germany) according to the manufacturer’s protocol. The RNA integrity was assessed with the Agilent 2100 Bioanalyzer system, which generates a RNA Integrity Number (RIN) [11].

### Complementary DNA synthesis

RNA (1 μg) was transcribed into complementary DNA (cDNA) in a final volume of 20 μl using the SuperScript VILO cDNA Synthesis Kit (Life Technologies, Darmstadt, Germany) according to the manufacturer’s protocol. The synthesis of cDNA was primed by using random oligodeoxyribonucleotides. The newly synthesised cDNA was diluted 10 times for subsequent use in RT-qPCR. Both diluted and undiluted cDNA were stored at -20°C until further use.

### Real-time-qPCR

The RT-qPCRs were performed using a StepOnePlus Real-Time PCR System (Life Technologies, Darmstadt, Germany) with TaqMan Gene Expression Assays. All primers, probes and TaqMan Fast Advanced Master Mix were purchased from Life Technologies (Darmstadt, Germany). The primers and probes used in this study are described in Table 1. Each reaction had a final volume of 20 μl and was set up as follow: 10 μl TaqMan Master Mix, 1 μl primer/probe mix (0.9 μM for the forward and reverse PCR primers and 0.25 μM for the probe), 4 μl diluted cDNA, 5 μl DNase/RNase free water. Each sample was loaded in duplicate and a negative control with all the components stated above except the cDNA was included on each reaction plate. Every reaction plate was repeated once again to ensure accuracy of results. The loaded MicroAmp Fast Optical 96-well Reaction Plate (Life Technologies, Darmstadt, Germany) was then centrifuged for 2 min at 1200 rpm prior to RT-qPCR. The thermal cycling method was set up according to the manufacturer’s protocol. Prior to gene expression analyses of the genes of interest (*MYC*, *Hif1α*, *CDX2*, *HMOX1* and *IL1β*), a screening was done to determine appropriate reference genes from a panel (*HPRT1*, *GUSB*, *PSMB6*, *RPL13*, *TBP*) using geNorm [12]. The relative gene expression was determined using the 2^-ΔΔC^_T_ method with the inclusion of a normalisation factor obtained by geNorm [12].

**Table 1:** Description of the TaqMan Gene Expression Assays used.

Gene	Accession number	Exon	Amplicon length (bp)	Dye /Quencher	Assay number
*HPRT1* (Reference gene)	NM_000194.2	1–2	72	VIC / MGB	Hs01003267_m1
*GUSB* (Reference gene)	NM_000181.3	8–9	96	VIC / MGB	Hs00939627_m1
*PSMB6* (Reference gene)	NM_001270481.1	2–3	93	VIC / MGB	Hs00382586_m1
*RPL13* (Reference gene)	NM_000977.3	6–6	137	VIC / MGB	Hs00744303_s1
*TBP* (Reference gene)	NM_001172085.1	2–3	91	VIC / MGB	Hs00427620_m1
*MYC* (Gene of interest)	NM_002467.4	1–2	87	FAM / MGB	Hs00905030_m1
*Hif1α* (Gene of interest)	NM_001530.3	1–2	62	FAM / MGB	Hs00936371_m1
*CDX2* (Gene of interest)	NM_001265.4	2–3	81	FAM / MGB	Hs01078080_m1
*HMOX1* (Gene of interest)	NM_002133.2	3–4	82	FAM / MGB	Hs01110250_m1
*IL1β* (Gene of interest)	NM_000576.2	3–4	91	FAM / MGB	Hs01555410_m1

### Statistical analyses

**Fig 2:**
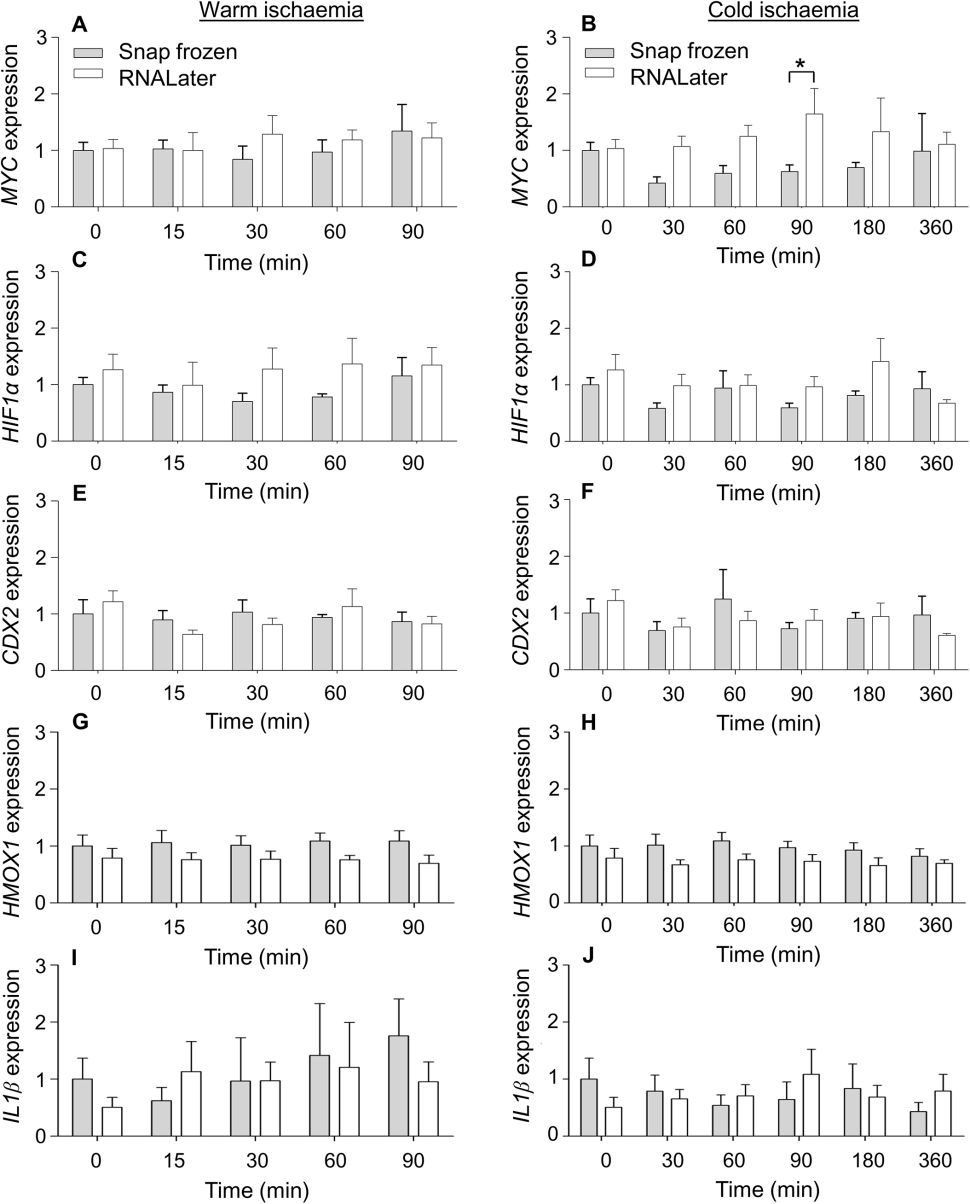
Normalised gene expressions of *MYC* (A, B), *HIF1α* (C, D), *CDX2* (E, F), *HMOX1* (G, H) or *IL1β* (I, J) after a time course of warm or cold ischaemia. Values represent means ± SEM. All values were *N* = 6 unless otherwise stated. The following values were *N* = 5; *HMOX1* expression at 0 and 90 min warm ischaemia (RNAlater) and 0 min cold ischaemia (RNAlater), *IL1β* expression at 90 min warm ischaemia (RNAlater) and 30 min cold ischaemia (snap frozen). The following values were *N* = 4; *IL1β* expression at 0 min warm or cold ischaemia (RNAlater). Four technical replicates were done per donor for each data point. *Significantly different from corresponding snap-frozen condition, *p* < 0.05.

**Fig 3:**
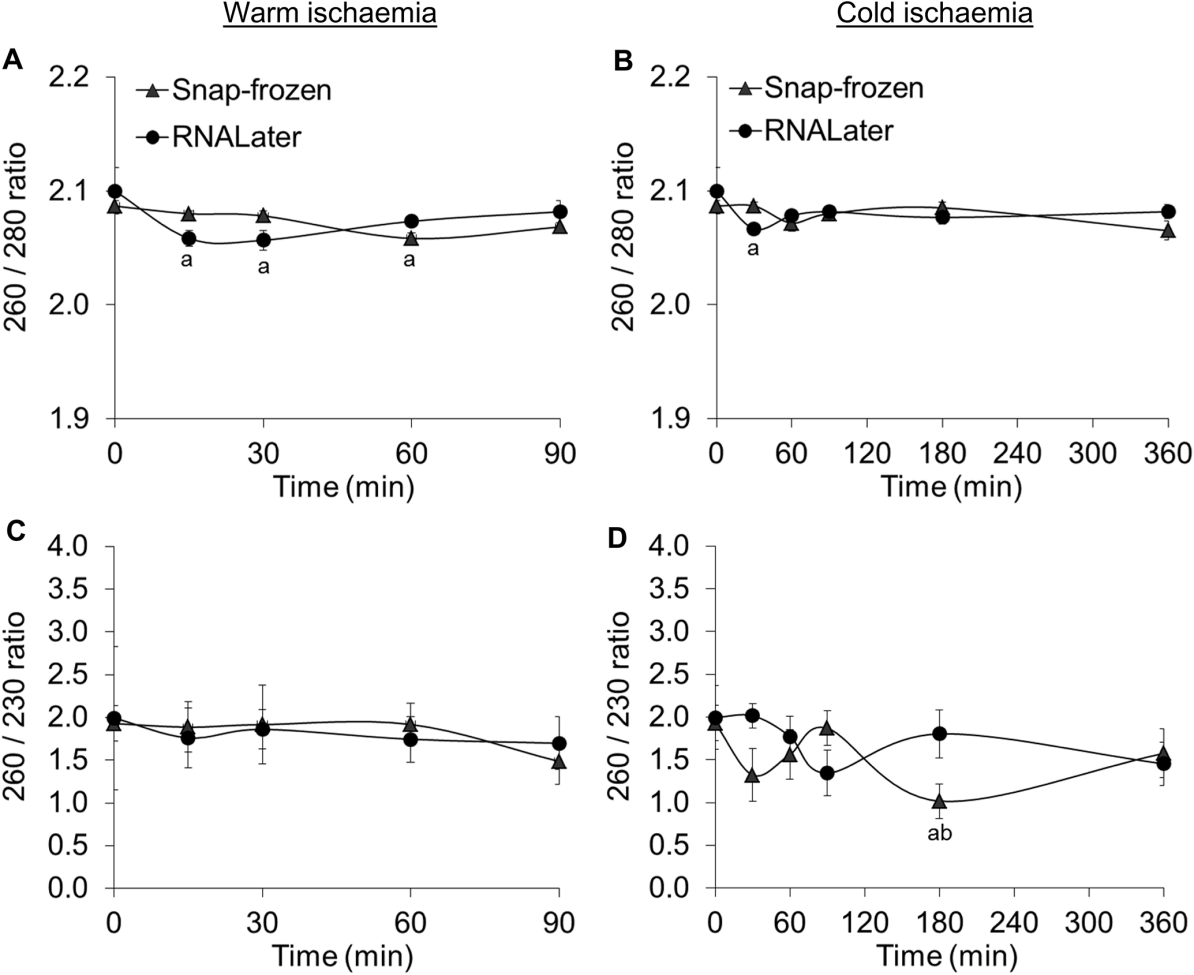
260/ 280 and 260/ 230 ratios after a time course of warm (A, C) or cold (B, D) ischaemia. Values represent means ± SEM. All values were *N* = 6 except for 3 values that were *N* = 5; 260/ 280 ratio for warm ischaemia at 30 min (snap frozen), 260/ 230 ratios for warm ischaemia at 0 min (RNAlater) and 30 min (snap frozen). ^a^Significantly different from corresponding 0 min condition, *p* < 0.05. ^b^Significantly different from corresponding 90 min condition, *p* < 0.05.

The data are represented as means ± standard error of the mean (SEM). For *MYC*, *Hif1α* and *CDX2* gene expressions obtained from 6 donors (Fig 2), statistical analyses were performed using a two-way ANOVA followed by an unprotected Fisher's Least Significant Difference (LSD) test when the two-way ANOVA showed significant changes. For Fig 3 and *HMOX1* and *IL1β* gene expressions in Fig 2 with different donor numbers, two-way ANOVAs were done with subsequent post tests performed using the Bonferroni method. Following statistical analyses, values were considered significantly different when the *p* value was less than 0.05. The statistical analyses were done using version 6 of Prism.

## Results

### Nucleic acid purity was sufficient for downstream experiments

260/280 ratios with values between 2 and 2.1 show that there is no protein contamination of the isolated RNA samples (Fig 3A and 3B). However, 260/ 230 ratios were below the range of 2 to 2.2, indicating that there may be residual chemical contamination from the RNA extraction procedure (Fig 3C and 3D). Thus, in order to avoid an overestimation of RNA concentration by spectrophotometric methods due to chemical contamination, RNA in solution was quantified using a fluorescent nucleic acid stain.

### The ileum mucosa retains tissue and cellular structure

**Fig 4:**
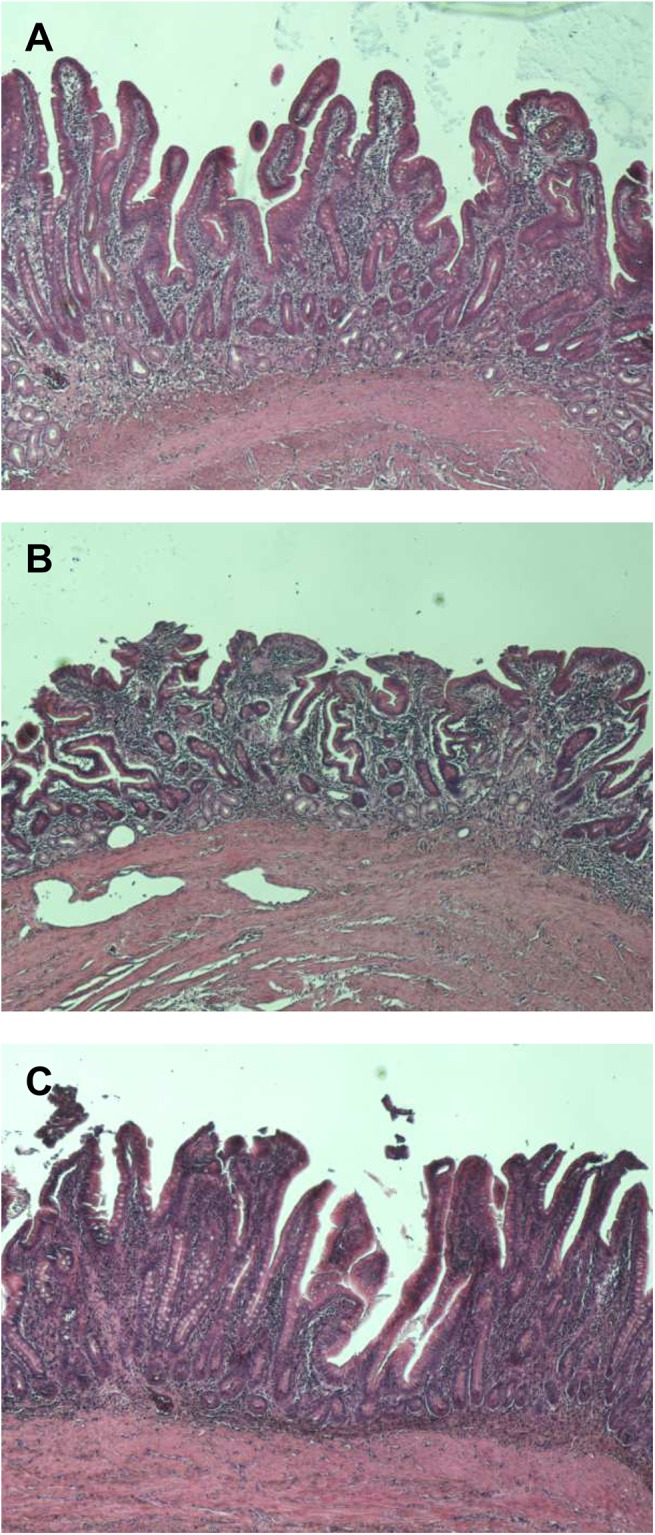
H & E staining of ileum section at 0 min (A), 90 min warm ischaemia (B) and 360 min cold ischaemia (C). The images were acquired at x 40 magnification.

H & E staining showed that the epithelium of the mucosa remains intact and the tissue retains characteristic tissue and cellular structure over the time course of warm or cold ischaemia (Fig 4).

### The ileum mucosa retains high RNA integrity after an extended period of warm and cold ischaemia

**Fig 5:**
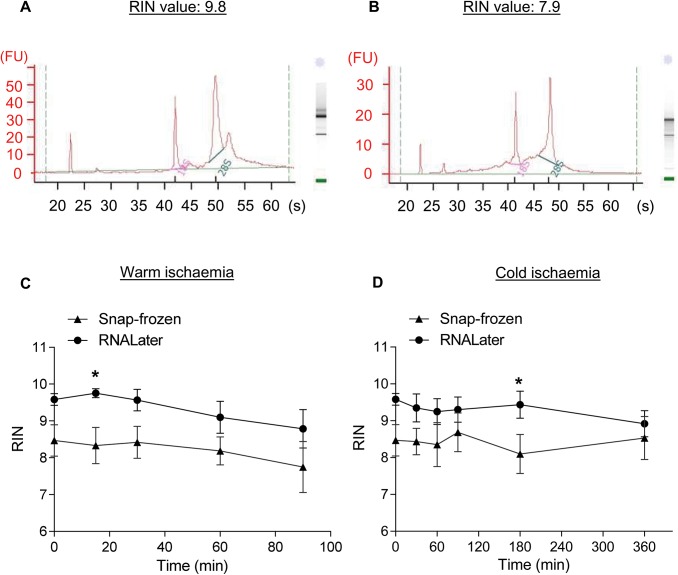
A representative electropherogram is shown for the highest (A) and lowest (B) average RNA Integrity Number (RIN) obtained in this study. RIN of ileum mucosa specimens collected by snap-freezing in liquid nitrogen or banked using RNA stabilization solution after a time course of warm (C) or cold ischaemia (D). Values represent means ± SEM with *N* = 6. *Significantly different from corresponding snap-frozen condition, *p* < 0.05.

The RNA integrity of each sample was assessed using the RIN assigned by the Bioanalyzer. Electropherograms representing the highest (9.8) and lowest (7.9) average RIN obtained in this study are shown in Fig 5A and 5B. When RIN was plotted against warm ischaemia time (Fig 5C) or cold ischaemia time (Fig 5D), there were generally no significant differences between snap-freezing or using RNA stabilization solution to bank the ileum mucosae over the time course. There were significant 1.2-fold increases in RIN when the tissue was banked using RNA stabilization solution only after 15 minutes of warm ischemia and 180 minutes of cold ischemia.

### Warm and cold ischaemia time does not affect gene expression in the ileum mucosa

**Fig 6:**
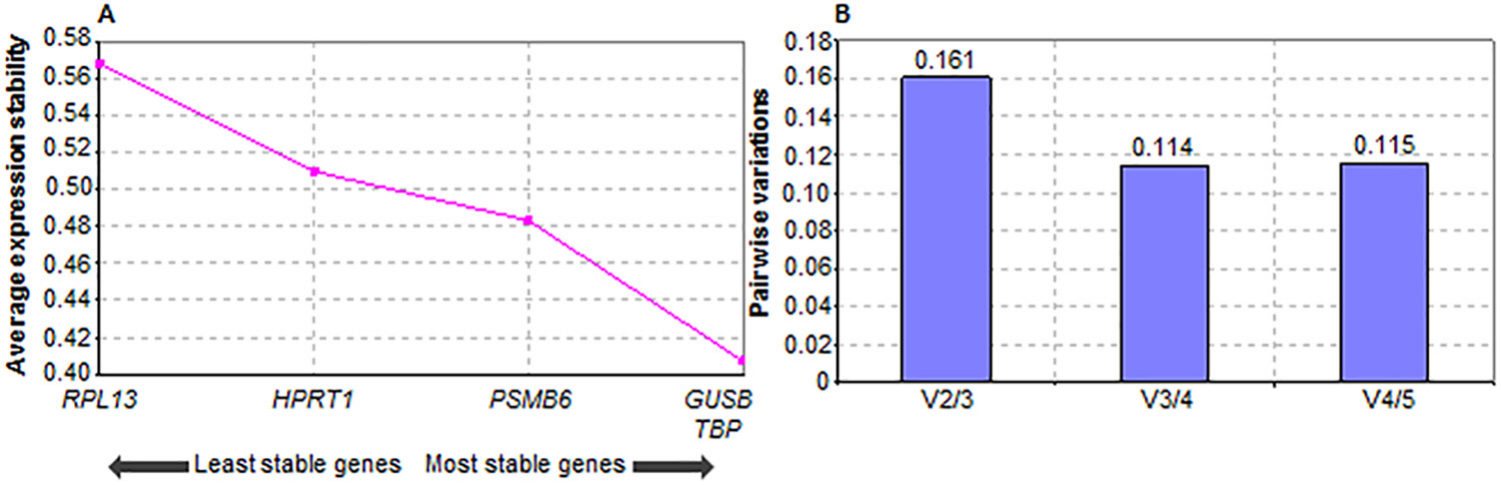
Genorm analysis to determine (A) average expression stability values of the reference genes during stepwise exclusion of the least stable reference gene and (B) pairwise variation analysis between the normalization factors to determine the number of reference genes required for accurate normalization. All values from 6 donors were used for the analysis with each gene expression assay done with 4 technical replicates.

Prior to gene expression analyses, suitable reference genes for this study had to be found. Therefore, 5 commonly used reference genes, *HPRT1*, *GUSB*, *PSMB6*, *TBP* and *RPL13*, were tested. GeNorm output indicated that *RPL13* was the least stable reference gene (Fig 6A) and suggested that four reference genes, *HPRT1*, *GUSB*, *PSMB6* and *TBP*, should be used to keep the pairwise variation less than 0.12 (Fig 6B). These four reference genes were sufficient as Vandesompele *et al*. [12] proposed that no additional reference gene is required when pairwise variations are less than 0.15.

To assess whether an extended period of warm or cold ischaemia affects gene expression in the ileum mucosa, the expressions of 5 genes of interest were examined. These genes include 4 inducible genes, which can respond to external stimuli by a rapid activation of gene expression and an intestine-specific gene (*CDX2*). In short, the 4 inducible genes examined included an immediate early gene (*MYC*), the hypoxia-inducible factor 1α (*HIF1α*), an inflammatory response gene (*IL1β*) and oxidative stress inducible gene (*HMOX1*). Real-time-qPCR analysis showed that none of these 5 genes showed significant differences in relative gene expression when compared across various time points for both warm and cold ischaemia (Fig 2). In addition, the processing of the ileum mucosa by snap-freezing or through the use of RNA stabilization solution for banking did not result in any significant differences except for a significant increase in the relative expression of *MYC* when banked using RNA stabilization solution after 90 min of cold ischemia by 2.6-fold (Fig 2B).

## Discussion

This study aimed to determine the effect of warm or cold ischaemia time on RNA integrity and gene expression in the ileum mucosa. Due to the nature of the surgery described in the method section above, ileum samples could be obtained in the operation room without warm ischaemia *in situ*. Ileum tissue was then sampled at various time-points after warm or cold ischaemia for comparison against the time 0 sample, which is a good representation of the *in vivo* condition (Fig 1).

RNA integrity is indicated by RIN, which is a number assigned by a software algorithm that ranges between 1 (totally degraded RNA) to 10 (fully intact RNA) [11]. Various publications have recommended minimum acceptable RINs after examining the relationship between RIN and the reliability of data obtained by downstream applications [13–18]. For RT-qPCR, Fleige *et al*. [14] stated that a RIN higher than 5 indicates good total RNA quality for amplification of PCR products up to 200 bp and a RIN higher than 8 indicates optimal total RNA for this application. However, Botling *et al*. [13] found that the critical RIN associated with potentially erroneous RT-qPCR expression values was below 8. This higher RIN requirement from Botling *et al*. [13] could be due to the use of oligo-dT primers for cDNA synthesis. For gene expression microarrays, authors have recommended RINs ≥ 6 [17], ≥7 [16, 18] or >5 (provided that genes that are short or have probe binding sites close to the 5’ end are excluded) [15]. In this study, the RINs (7.8±0.6–9.8±0.1) obtained from all time-points, temperatures or banking methods indicate that the RNA isolated from the ileum mucosa is of a very high quality. Thus, these RNA samples should be of a sufficient quality for downstream analyses using RT-qPCR or gene expression microarrays. It is interesting to note that such high RIN can be obtained despite the expression of RNases in the gut [6–9]. This could be due to tissue and cellular structure being maintained at these early stages (Fig 4), which would restrict exogeneous RNase access to intracellular RNA [3, 13].

With regard to the RIN, S1 Table summarises the effect of time, temperature and banking method on this number. In short, it has been found that the use of RNA stabilization solution generally results in higher RIN compared to snap-freezing [19–21]. Similarly, this study found that significantly higher RINs (1.2-fold) were obtained for 2 of the examined time-points when the samples were banked in RNA stabilization solution instead of being snap-frozen. In contrast, although the samples banked in RNA stabilization solution tended to have higher RINs, there were in general no significant differences between the 2 banking methods. As samples here were snap-frozen or placed in RNA stabilization solution at the same time using the same dissection technique, this discrepancy could be due to the nature of the banking method itself. Both methods preserve RNA through the prevention of RNase activity either by sub-zero temperatures or by precipitating RNases and RNA. However, for snap-frozen samples, enzymatic activity is only temporarily inactivated by the low temperature. If the sample is thawed, this process destroys tissue and cellular structure allowing RNases to commence degradation of RNA as the temperature rises [13, 18]. As such, great care must be taken when processing the sample for subsequent steps to prevent RNA degradation and to obtain comparable RNA quality.

Further, some authors have found that there were no significant differences in RIN over a time course in human colon cancers (up to 4 h) [22], rectal and distal sigmoid tumours (up to 2 h) [19], pancreatic tumours (up to 1 h) [16] or mouse skin (up to 1 h) [23] (S2 Table). However, Hong *et al*. [24] found that RIN in human colorectal cancers decreased with time (S1 Table). This study has results similar to the first group; RINs in human ileum mucosa were not significantly different for up to 3 h of warm ischaemia or 6 h of cold ischaemia. As stated above, RNA integrity appears to be maintained in intact tissue. It is possible that RIN can be decreased in tissues subjected to trauma during surgery or dissection [20] or in samples with necrosis [13].

For gene expression, there was only a significant increase in the relative expression of *MYC* after 90 minutes of cold ischaemia when banked using RNA stabilization solution. This result could be due to cold ischaemia triggering a transient overexpression of *MYC* at 90 minutes. However, this scenario is not very likely as a corresponding increase in *MYC* expression should have occurred in the snap-frozen samples. Instead, this increase could be due to an inadvertent sampling of cancerous tissue at this time-point, although the tissue obtained is from the resection margin, as it is known that colorectal carcinomas frequently have overexpression of MYC [25, 26]. Other than this significant value, there are in general no significant changes in gene expression. Similarly, the studies done on a wide variety of tissues show that although there were changes in gene expression with time and banking method, the majority of the genes examined did not have significantly different expression levels (S2 Table) [4, 19, 21, 23, 27–31]. Considering that many of the above studies were done with microarrays, which in many cases have a 3’ bias due to the requirement for mRNAs with a poly-A tail and subsequent hybridization to a probe that may bind at some distance from the 3’ end [5, 32], the gene expression profiles obtained were surprisingly stable. Thus, this can explain why gene expression in ileum mucosa is stable as RT-qPCR is more robust especially when random hexamers are used for reverse transcription of cDNA, amplicon lengths are short and suitable reference genes are chosen [14].

In conclusion, ileum mucosa has a high RNA integrity and stable gene expression over a time course of warm or cold ischaemia whether banked in RNA stabilization solution or snap-frozen in liquid nitrogen. Since the average warm and cold ischaemia times imposed by surgery and the process of tissue banking is shorter than what was examined in this study, human ileum mucosa samples could be used for gene expression studies. Nonetheless, it is important to check that one’s genes of interest are not in the small proportion of genes affected by such conditions before proceeding. As to whether snap-freezing or banking in RNA stabilization solution should be done, both methods offer different advantages and disadvantages. RNA stabilization solution offers ease of use compared to liquid nitrogen and is also more operator-friendly compared to snap-frozen samples, which should not be thawed at all during processing steps. On the flip side, snap-frozen samples offer more versatility as samples banked using RNA stabilization solution have been found to provide poor microscopic images due to a loss in tissue morphology [13, 33] and have been found to be unsuitable for immunohistochemistry [33].

## Supporting Information

S1 Table The effect of time, temperature (°C) and banking methods on the RNA integrity number (RIN). (DOCX)

S2 Table The effect of time and banking methods on gene expression. (DOCX)
