# Supplementary material for: Pre-Analytical Determination of the Effect of Extended Warm or Cold Ischaemia on RNA Stability in the Human Ileum Mucosa
Source: PLoS One. 2015 Sep 15;10(9):e0138214. doi: 10.1371/journal.pone.0138214 (PMC4570714; doi:10.1371/journal.pone.0138214)
Supplement: S1 Table — (DOCX) [file pone.0138214.s001.docx]

**Table S1. The effect of time, temperature (°C) and banking methods on the RNA integrity number (RIN).**

| Species | Tissue | Time points | °C | Banking method | Significant changes in RIN | N | Ref |
| --- | --- | --- | --- | --- | --- | --- | --- |
| Human | First trimester placenta | 0*, 30*, 60* and 120* min | RT | RNAlater | *Increased compared to corresponding snap-frozen samples | 4 | [[1](#_ENREF_1)] |
| Human | Third trimester placenta | 0*, 30*, 60* and 120 min | RT | RNAlater | *Increased compared to corresponding snap-frozen samples | 4 | [[1](#_ENREF_1)] |
| Human | Lung cancer | Immediately after diagnostic biopsy* | - | RNAlater | *Increased compared to corresponding snap-frozen samples | 3 | [[2](#_ENREF_2)] |
| Human | Colon cancer | 0.5, 1, 2 or 4 h after surgical resection | On ice/ RT | Snap-frozen | No significant differences between time-points | 5 | [[3](#_ENREF_3)] |
| Human | Rectal and distal sigmoid tumours | 0 min after biopsy immediately prior to surgical resection. | - | RNAlater | Increased compared to samples snap-frozen at 0, 15, 30, 60 and 120 min. | 10 | [[4](#_ENREF_4)] |
| Human | Rectal and distal sigmoid tumours | 0, 15, 30, 60 and 120 min after biopsy immediately prior to surgical resection. | - | Snap-frozen | No significant differences between all time-points | 10 | [[4](#_ENREF_4)] |
| Human | Pancreatic tumour | ≤10, 11-30, 31-60 or >60 min procurement time after surgery | - | Snap-frozen | Proportion of samples with RIN ≥ 7 not significantly different between the time points | 12 | [[5](#_ENREF_5)] |
| Mouse | Skin | 0, 5, 10, 15, 20, 25, 30, 45 and 60 min post-mortem | RT | RNAlater | No significant differences between all time-points | 3 | [[6](#_ENREF_6)] |
| Human | Colorectal cancer | 10, 30^, 60^, 90^ min after surgical extraction | On ice | Snap-frozen | ^Decreased compared to 10 min condition | 20 | [[7](#_ENREF_7)] |
| Human | Liver | 0 h, after operation, after pathological examination (0 h, 3 h and 1 d) | On ice/ RT | Snap-frozen/ RNAlater | Generally no differences for all data points, except for a decrease 1 d after pathology at RT and a slight increase using RNAlater at two time points. | 6 | [[8](#_ENREF_8)] |

Abbreviations; Room temperature (RT)

**References**

1. Wolfe LM, Thiagarajan RD, Boscolo F, Tache V, Coleman RL, et al. (2014) Banking placental tissue: an optimized collection procedure for genome-wide analysis of nucleic acids. Placenta 35: 645-654.

2. Lawson MH, Rassl DM, Cummings NM, Russell R, Morjaria JB, et al. (2010) Tissue banking of diagnostic lung cancer biopsies for extraction of high quality RNA. J Thorac Oncol 5: 956-963.

3. Bao WG, Zhang X, Zhang JG, Zhou WJ, Bi TN, et al. (2013) Biobanking of fresh-frozen human colon tissues: impact of tissue ex-vivo ischemia times and storage periods on RNA quality. Ann Surg Oncol 20: 1737-1744.

4. Bray SE, Paulin FE, Fong SC, Baker L, Carey FA, et al. (2010) Gene expression in colorectal neoplasia: modifications induced by tissue ischaemic time and tissue handling protocol. Histopathology 56: 240-250.

5. Rudloff U, Bhanot U, Gerald W, Klimstra DS, Jarnagin WR, et al. (2010) Biobanking of human pancreas cancer tissue: impact of ex-vivo procurement times on RNA quality. Ann Surg Oncol 17: 2229-2236.

6. Gopee NV, Howard PC (2007) A time course study demonstrating RNA stability in postmortem skin. Exp Mol Pathol 83: 4-10.

7. Hong SH, Baek HA, Jang KY, Chung MJ, Moon WS, et al. (2010) Effects of delay in the snap freezing of colorectal cancer tissues on the quality of DNA and RNA. J Korean Soc Coloproctol 26: 316-323.

8. Lee SM, Schelcher C, Gashi S, Schreiber S, Thasler RM, et al. (2013) RNA stability in human liver: comparison of different processing times, temperatures and methods. Mol Biotechnol 53: 1-8.
