# Supplementary material for: Pre-Analytical Determination of the Effect of Extended Warm or Cold Ischaemia on RNA Stability in the Human Ileum Mucosa
Source: PLoS One. 2015 Sep 15;10(9):e0138214. doi: 10.1371/journal.pone.0138214 (PMC4570714; doi:10.1371/journal.pone.0138214)
Supplement: S2 Table — (DOCX) [file pone.0138214.s002.docx]

**Table S2. The effect of time and banking methods on gene expression.**

| Species | Tissue | Time points | °C | Banking method | Significant changes in gene expression | Method | N | Ref |
| --- | --- | --- | --- | --- | --- | --- | --- | --- |
| Human | First trimester placenta | 0 or 120 min after delivery | RT | RNAlater/ Snap-frozen | 95-98% of probes not different between time-points (27719 probes in total) | Oligonucleotide microarray | 3 | [[1](#_ENREF_1)] |
| Human | Third trimester placenta | 0 or 120 min after extraction | RT | RNAlater/ Snap-frozen | 95-99% of probes not different between time-points (27719 probes in total) | Oligonucleotide microarray | 3 | [[1](#_ENREF_1)] |
| Human | Breast carcinoma | 0.5 or 18-24 h after surgical excision | 4°C | Snap-frozen | Relative levels of 190 individual mRNAs not different between time-points | cDNA microarray | 1 | [[2](#_ENREF_2)] |
| Human | Primary breast tumours | 0, 2, 6, and 24 h after resection. | RT | Snap-frozen | ~96% of genes not different (17172 probes). 0.4, 0.7 and 1.8% of genes upregulated at 2, 6 and 24 h respectively. 0.4, 0.3 and 2.3% of genes downregulated at 2, 6 and 24 h respectively. | cDNA microarray/ RT-qPCR (4 genes for confirmation) | 8 | [[3](#_ENREF_3)] |
| Human | Rectal and distal sigmoid tumours | 0 min after biopsy immediately prior to surgical resection. | - | RNAlater/ snap-frozen | 77% not different (less than 2-fold change) (~23478 probes in total) | Oligonucleotide microarray | 3 | [[4](#_ENREF_4)] |
| Human | Rectal and distal sigmoid tumours | 0, 15, 30, 60 and 120 min after biopsy immediately prior to surgical resection. | - | Snap-frozen | 70% not different (less than 2-fold change) over the course of 120 min (~23478 probes in total) | Oligonucleotide microarray/ RT-qPCR (4 genes for confirmation) | 3 | [[4](#_ENREF_4)] |
| Human | Prostate peripheral zone | Biopsies during in situ or *ex situ* period | - | RNAlater | 92% not different. 8% increased (91 genes of interest in total) | RT-qPCR array/ single RT-qPCR (4 genes for confirmation | 10 | [[5](#_ENREF_5)] |
| Human | Colon | 5 min or 30 min after colon resection | - | Snap-frozen | 20% showed difference in expression (>2-fold) between the time-points (11984 probes) | Oligonucleotide microarray | 2 | [[6](#_ENREF_6)] |
| Mouse | Skin | 0, 5, 10, 15, 20, 25, 30, 45 and 60 min post-mortem | RT | RNAlater | No significant differences between all time-points | RT-qPCR (4 genes) | 3 | [[7](#_ENREF_7)] |
| Human | Prostate cancer | *In situ* biopsy core after anaesthesia or *ex situ* biopsy after removal | - | Snap-frozen | Surgical manipulation increases transcript levels in 2.3% of the cDNAs using a criterion of 15% FDR | cDNA microarray / RT-qPCR (3 genes for confirmation) | 12 | [[8](#_ENREF_8)] |
| Human | Liver | 0 h, after operation, after pathological examination (0 h, 3 h and 1 d) | On ice/ RT | Snap-frozen/ RNAlater | Generally no significant differences between all time-points | RT-qPCR (4 genes) | 6 | [[9](#_ENREF_9)] |

Abbreviations; Room temperature (RT), False-discovery rate (FDR).

**References**

1. Wolfe LM, Thiagarajan RD, Boscolo F, Tache V, Coleman RL, et al. (2014) Banking placental tissue: an optimized collection procedure for genome-wide analysis of nucleic acids. Placenta 35: 645-654.

2. Sewart S, Barraclough R, Rudland PS, West CR, Barraclough DL (2009) Molecular analysis of a collection of clinical specimens stored at 4 degrees C as an alternative to snap-freezing. Int J Oncol 35: 381-386.

3. De Cecco L, Musella V, Veneroni S, Cappelletti V, Bongarzone I, et al. (2009) Impact of biospecimens handling on biomarker research in breast cancer. BMC Cancer 9: 409.

4. Bray SE, Paulin FE, Fong SC, Baker L, Carey FA, et al. (2010) Gene expression in colorectal neoplasia: modifications induced by tissue ischaemic time and tissue handling protocol. Histopathology 56: 240-250.

5. Schlomm T, Nakel E, Lubke A, Buness A, Chun FK, et al. (2008) Marked gene transcript level alterations occur early during radical prostatectomy. Eur Urol 53: 333-344.

6. Spruessel A, Steimann G, Jung M, Lee SA, Carr T, et al. (2004) Tissue ischemia time affects gene and protein expression patterns within minutes following surgical tumor excision. Biotechniques 36: 1030-1037.

7. Gopee NV, Howard PC (2007) A time course study demonstrating RNA stability in postmortem skin. Exp Mol Pathol 83: 4-10.

8. Lin DW, Coleman IM, Hawley S, Huang CY, Dumpit R, et al. (2006) Influence of surgical manipulation on prostate gene expression: implications for molecular correlates of treatment effects and disease prognosis. J Clin Oncol 24: 3763-3770.

9. Lee SM, Schelcher C, Gashi S, Schreiber S, Thasler RM, et al. (2013) RNA stability in human liver: comparison of different processing times, temperatures and methods. Mol Biotechnol 53: 1-8.
